# 35.4 A PUBLIC HEALTH APPROACH TO THE PREVENTION OF PSYCHOSIS

**DOI:** 10.1093/schbul/sby014.150

**Published:** 2018-04-01

**Authors:** Robin Murray, Marta Di Forti, Evangelos Vassos, Antonella Trotta, Harriet Quigley, Olesya Ajnakina, Diego Quattrone, Giada Tripoli, Victoria Rodriguez, Craig Morgan

**Affiliations:** Institute of Psychiatry, Psychology & Neuroscience, King’s College London

## Abstract

**Background:**

The main attempt to prevent the development of psychosis has been through clinics for people at clinical high risk. Such an approach is useful for research but can never reach the majority of individuals who will become psychotic. Biological markers could be used to identify individuals with unusual vulnerabilities e.g. those with copy number variations such as VCFS. However, identifying the with such markers is unlikely to impact on the majority of cases, and as yet no useful interventions are available. How therefore to prevent psychosis?

**Methods:**

Data will be presented from 3 studies of first onset psychosis (FEP) which used similar methods of ascertainment and assessment of cases and controls; AESOP and GAP from South London and the EU-GEI across 16 sites in 5 European countries.

**Results:**

The identified risk factors for psychosis were the polygenic risk score for schizophrenia, childhood abuse, living in a city, being from an ethnic minority, drug abuse, adverse life events. Clearly, reducing some of these (e.g. urbanicity or migration) is not within the powers of psychiatrists. The GAP study showed that the polygenic risk score accounted for the greatest variance in caseness; those with scores in the highest quintile were 7 times more likely to be a psychotic case than those in those lowest quintile. The GAP study also gave estimates of the population attributable fraction (PAF): these indicated that if no one was exposed to child abuse and use of high potency cannabis, then 16% and 24% respectively of psychosis in South London could be prevented. The EU-GEI study showed striking differences in the incidence of psychosis between Northern and Southern Europe; data will be prevented concerning the contribution of risk factors, especially cannabis use, to this.

**Discussion:**

The knowledge that schizophrenia is the extreme of a continuum of psychosis has important implications for prevention. Preventive approaches to hypertension or obesity do not focus on identifying individuals carrying biological markers; rather they encourage members of the general population to take exercise and reduce their calorie intake. A similar approach should be adopted for psychosis. In the long-term attempts to reduce risk factors should be made e.g. addressing psychotogenic aspects of city living or by decreasing discrimination of ethnic minorities. This will be difficult. However, an obvious place to start is by attempting to influence society’s patterns of consumption of high-potency cannabis. Unfortunately, public policy in the US and certain other countries appears to be moving in the opposite direction with increases in consumption and potency. Are these countries sleep-walking to more psychosis?

